# Cationic Silicon Nanocrystals with Colloidal Stability, pH‐Independent Positive Surface Charge and Size Tunable Photoluminescence in the Near‐Infrared to Red Spectral Range

**DOI:** 10.1002/advs.201500263

**Published:** 2016-01-21

**Authors:** Kenneth K. Chen, Kristine Liao, Gilberto Casillas, Yiying Li, Geoffrey A. Ozin

**Affiliations:** ^1^ Department of ChemistryUniversity of Toronto80 St. George StreetTorontoOntarioM5S 3H6Canada; ^2^ UOW Electron Microscopy CentreUniversity of WollongongWollongongNew South Wales2500Australia; ^3^ Department of Materials Science & EngineeringUniversity of Toronto184 College Street, Suite 140TorontoOntarioM5S 3E4Canada

**Keywords:** silicon nanocrystals, silicon nanocrystal cations, silicon quantum dots

## Abstract

In this report, the synthesis of a novel class of cationic quaternary ammonium‐surface‐functionalized silicon nanocrystals (ncSi) using a novel and highly versatile terminal alkyl halide‐surface‐functionalized ncSi synthon is described. The distinctive features of these cationic ncSi include colloidal stability, pH‐independent positive surface charge, and size‐tunable photoluminescence (PL) in the biologically relevant near‐infrared‐to‐red spectral region. These cationic ncSi are characterized via a combination of high‐resolution scanning transmission electron microscopy with energy‐dispersive X‐ray analysis, Fourier transform infrared, X‐ray photoelectron, and photoluminescence spectroscopies, and zeta potential measurements.

## 1 Introduction

Quantum‐confined semiconductor nanocrystals exhibit size tunable optical properties, which are desirable for biological fluorescent‐labeling applications.^1^, ^2^ Despite many advantages over established fluorescent dye systems, the increased complexity of interactions with biological systems introduced by the relatively large size, varying core, and surface chemical and physical properties, and safety concerns, have prevented large‐scale adoption of these nanocrystal fluorophores.^3^ In particular, the toxicity of heavy‐metal‐based nanocrystals has been subject to intense scrutiny.^4^, ^5^, ^6^, ^7^ In recent years, silicon nanocrystals (ncSi) have been proposed and demonstrated as potentially nontoxic alternatives to established II–VI (e.g., CdSe/Te, ZnS/Se), III–V (e.g., InP), I–III–VI (e.g., CuInS/Se), and IV–VI (e.g., PbS/Se) nanocrystal systems for fluorescent labeling.^4^, ^8^, ^9^, ^10^

In addition to its nontoxic nature, the size of ncSi can be tailored to enable it to emit photoluminescence (PL) in the 650–900 nm spectral range, a window with minimal absorption from tissue components. Moreover, ncSi can be covalently bonded to a protective and water‐soluble coating of ligands via hydrosilylation.^11^, ^12^ Strategies for increased biocompatibility and aqueous stabilization can be generalized into two categories, namely steric and electrostatic. Steric stabilization strategies typically use long poly(ethylene glycol) chains to achieve colloidal stability. The use of solid lipid nanoparticles by Henderson et al. and phospholipid micelles by Erogbogbo et al. are prime examples.^13^, ^14^, ^15^ Electrostatic stabilization strategies rely on ionic ligands to prevent flocculation, commonly used ones include olefins with terminal carboxylic acids and terminal primary amines.^8^, ^12^, ^15^, ^16^, ^17^, ^18^, ^19^, ^20^ Korgel and co‐workers demonstrated a strategy using amphiphilic polymers to encapsulate ncSi in 2010.^21^

When selecting the appropriate surface functionalization strategy for a targeted application such as fluorescent labeling, key parameters that must be considered are hydrodynamic diameter, pH‐dependent stability, and optical properties. For example, ncSi based on steric stabilization strategies typically have larger hydrodynamic diameters ranging from 20 to 100 nm, and incorporates many ncSi in one nanoparticle, whereas ncSi based on electrostatic strategies typically have diameters below 5 nm and are dispersible as isolated nanocrystals.^12^, ^15^, ^17^, ^21^ In general, hydrodynamic diameters below about 5 nm are desirable since nanoparticles below this threshold can be cleared through urinary excretion to minimize potential toxicity effects.^4^

The cellular uptake mechanism of ncSi is not well understood and likely depends strongly on its surface chemistry. However, in many cases the nanoparticles ultimately gather in the lysosomes of cells, which have significantly lower pH (pH 5) than the cytoplasm (pH 7).^22^, ^23^ The fluctuating pH conditions within biological systems do not strongly affect strategies relying on steric stabilization. However, current electrostatic stabilization strategies can be strongly affected by pH and cause undesirable agglomeration of nanoparticles due to a loss of surface charge when carboxylate groups or amine groups are respectively protonated or deprotonated.^12^, ^24^ Therefore, to maintain pH‐independent solution stability while retaining the small size of ncSi, one must establish pH‐independent charge on the surface of the ncSi. To the best of our knowledge, this has not been achieved in any previously reported ncSi systems.

As a step towards this goal, we have developed a versatile alkyl halide‐functionalized ncSi platform, which not only protects the ncSi against oxidation by providing good surface coverage with alkyl chains, but also offers the opportunity to carry out a wide variety of organic reactions on the surface of these nanoparticles.^25^, ^26^ By installing a judiciously selected reactive site on the ligand terminus, we can prevent any possible side reactions between desired functional groups with the hydride‐functionalized ncSi precursor. This also allows us to avoid degradation of the desired functional groups due to the challenging conditions of the hydrosilylation reaction. With the reactive terminal alkyl halide‐ functionalized ncSi, it is possible to then install various useful functional groups, such as phosphonates, sulfonates, quaternary ammonium, and phosphonium cations, molecular dyes, amino acids on the surface of ncSi. In this report, we will demonstrate the flexibility of this prototype platform with the synthesis of colloidally stable, pH insensitive, NIR‐emitting, quaternary ammonium‐functionalized ncSi cations.

We selected the quaternary ammonium cation group for two reasons. First, it provides a pH‐independent positive charge on the ncSi surface, which will improve its aqueous dispersibility. Second, we wished to demonstrate the creation of the first ncSi‐amine system, which emit in the biologically significant NIR‐to‐red spectral region.^27^

It is worth noting that amines have a long history of strongly influencing the PL wavelength, excited‐state lifetime, and PL mechanism of silicon nanostructures. Despite its long history, the origin of these effects has been subject to much debate. For example, PL quenching in ncSi by primary alkyl amines, mainly attributed to dative surface‐bonded adducts, have been observed by Rho et al.^28^, ^29^ Furthermore, ncSi with Si–N–R linkages (where R = alkyl or aryl groups) normally have blue or green emission with excitation wavelength‐dependent PL and nanosecond excited‐state lifetimes. These effects were tentatively attributed to surface‐mediated charge transfer processes.^30^, ^31^ Previous reports of ncSi with Si–R–NH_2_ linkages (where R = alkyl or alkenyl groups) also emit in the blue region. Work in this area by Shiohara et al., Zuilhof et al., and Veinot et al. all report excitation wavelength‐dependent blue PL and nanosecond excited‐state lifetimes, which were attributed to direct bandgap transitions.^18^, ^20^, ^32^ In contrast to these blue and green emitting ncSi‐amine systems, alkyl‐ and allyl‐benzene‐passivated ncSi normally exhibit microsecond excited‐state lifetimes attributed to indirect bandgap transitions, excitation wavelength‐independent PL, and size‐dependent PL exhibiting strong quantum confinement effects.^30^, ^33^, ^34^ These contrasting optical properties seem to point to two distinct PL mechanisms: one which is core‐mediated, with long excited‐state lifetimes, and excitation wavelength‐independent PL, and one which is surface‐mediated, with short excited‐state lifetimes, and excitation wavelength‐dependent PL. Our demonstration of the first ncSi‐amine system, which show core‐mediated and size‐tunable PL can help shed new light on this decade long debate.

This is an open access article under the terms of the Creative Commons Attribution License, which permits use, distribution and reproduction in any medium, provided the original work is properly cited.

## 2 Results and Discussion

### 2.1 Synthesis of Alkyl Halide‐ and Quaternary Ammonium‐Passivated ncSi

**Figure 1:**
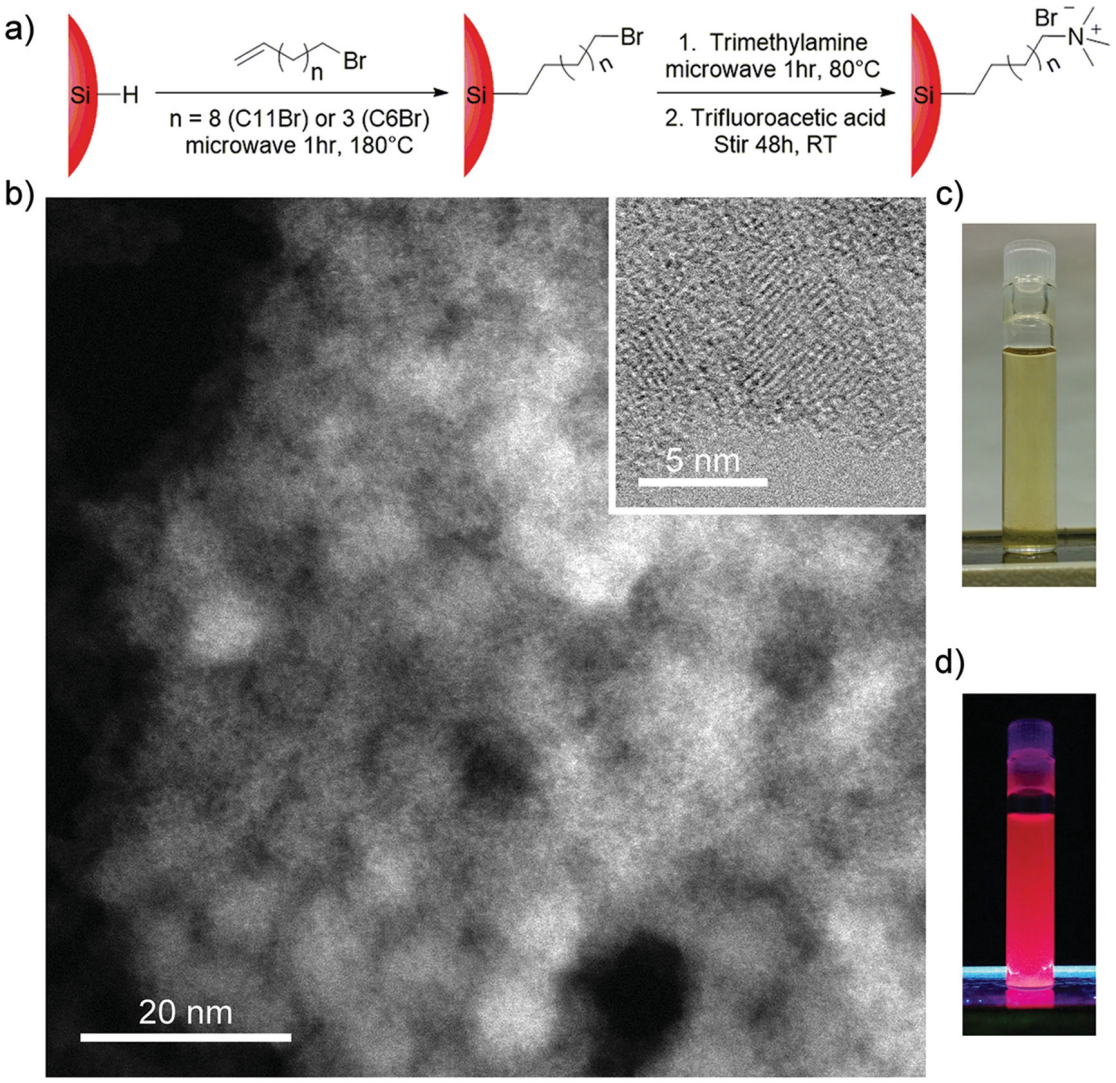
a) Synthetic scheme of ncSi:C11TMA and ncSi:C6TMA. b) MAADF STEM images of ncSi:C11Br with inset bright‐field STEM image showing lattice fringes. Images showing a typical ncSi:C11TMA sample in ethanol solution under c) visible and d) UV illumination.

Hydride‐terminated ncSi (ncSi:H) were prepared from thermally disproportionated silicon monoxide powder etched in aqueous hydrofluoric acid. The ncSi:H was dispersed in 11‐bromo‐1‐undecene or 6‐bromo‐1‐hexene and hydrosilylation was carried out under microwave irradiation to form ncSi:C11Br and ncSi:C6Br, respectively. Once purified from free ligands, the alkyl halide‐passivated ncSi was stirred with excess trimethylamine (TMA) to form ncSi:C11TMA and ncSi:C6TMA (**Figure** **1**a). The solution was then treated with excess trifluoroacetic acid (TFA) and purified by repeated precipitation and re‐dispersion via sonication washes (see details on the synthetic procedure in the Experimental Section). To ensure that the experimental quaternization conditions do not lead to significant side reactions such as hydroxylation or elimination, an analogous ligand‐only reaction was conducted. The ^1^H NMR spectrum of 11‐bromo‐1‐undecene before and after treatment with TMA show the quaternary ammonium cation being the only product, demonstrating that no side reactions of the ligand take place (Figure S1, Supporting Information).

### 2.2 Characterization of Alkyl Halide‐ and Quaternary Ammonium‐Passivated ncSi

The ncSi:C11Br and ncSi:C11TMA were characterized via middle angle annular dark field scanning transmission electron microscopy (MAADF‐STEM) (Figure 1b, Figure S2, Supporting Information). The sizes of the nanoparticles were approximately 4.8 ± 0.8 nm and 4.7 ± 0.9 nm in diameter for ncSi:C11Br and ncSi:C11TMA, respectively. These dimensions are close to those of NIR‐emitting alkyl‐passivated ncSi previously reported by Sun et al.^35^ Bright‐field STEM was used to better observe the lattice fringes of the nanoparticles, which showed 0.31 nm lattice constants characteristic of ncSi.^33^ Energy‐dispersive X‐ray spectroscopy (EDX) was used to evaluate the levels of silicon, carbon, nitrogen, and bromine in ncSi:C11Br and ncSi:C11TMA (Figure S3, Supporting Information). Both samples showed the expected high levels of silicon and carbon and presence of bromine. We observe a significant increase in levels of nitrogen in ncSi:C11TMA, which demonstrate successful quaternization.

**Figure 2:**
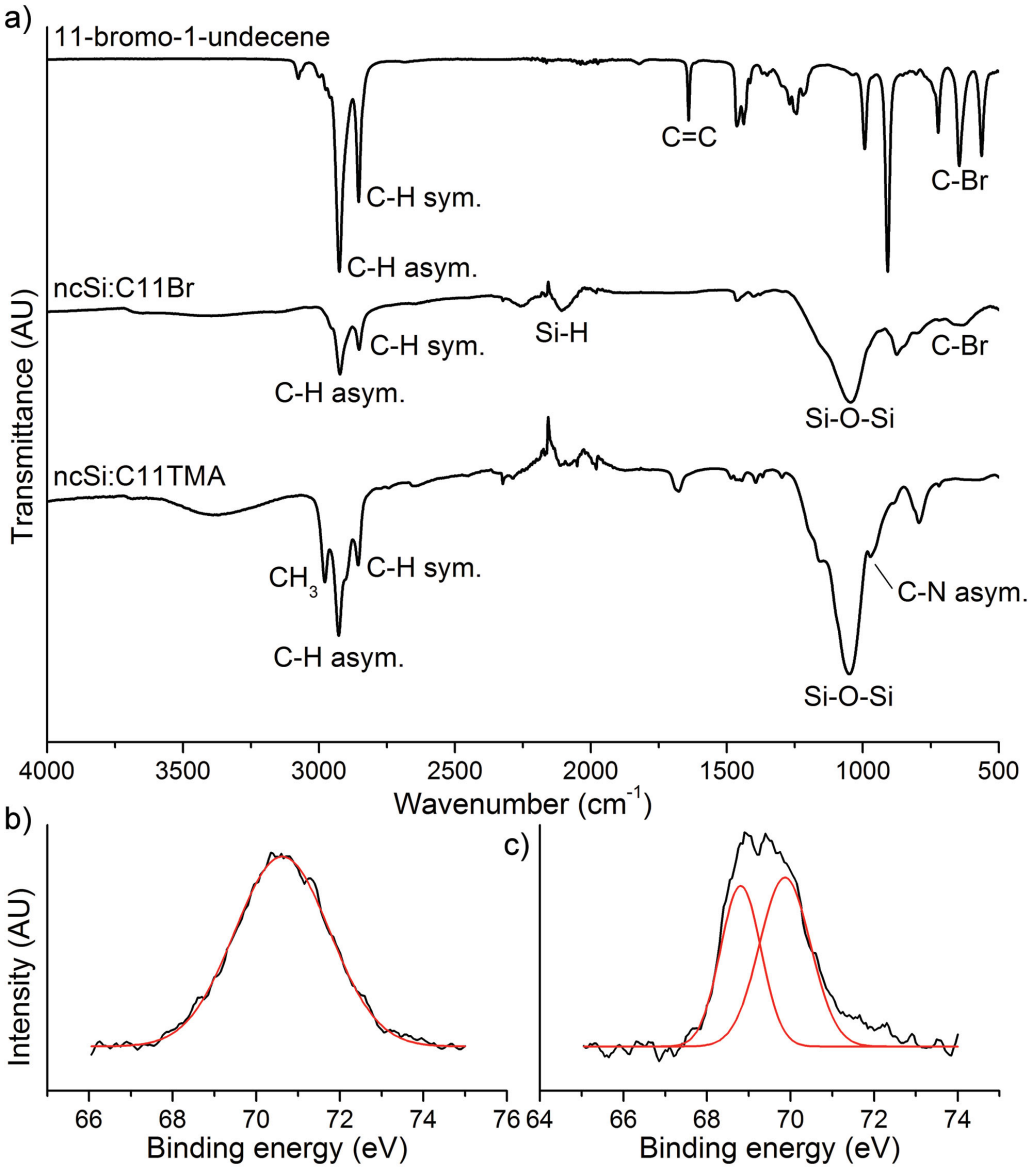
a) ATR‐FTIR spectra of 11‐bromo‐1‐undecene, ncSi:C11Br, and ncSi:C11TMA (from top to bottom) with significant vibrational modes labeled. High‐resolution XPS scan of b) ncSi:C11Br and c) ncSi:C11TMA show the shift in binding energy of the Br 3d peaks.

Successful hydrosilylation of ncSi:H and retention of the alkyl halide group is assessed using attenuated total reflectance infrared spectroscopy (ATR‐FTIR). Thermally initiated hydrosilylation is generally accepted to follow a radical mechanism, which may react with the alkyl bromide group and result in “inverted” ligands with outward‐facing terminal alkenes. This is not the case for our synthesis, as shown in **Figure** **2**a, the vibrational mode corresponding to a C=C stretch (1640 cm^−1^) characteristic of 11‐bromo‐1‐undecene completely disappears after hydrosilylation to form ncSi:C11Br. We also observe the presence of remnant Si–H (2107 cm^−1^) and Si–O–Si (1045 cm^−1^) stretches characteristic of ncSi. The appearance of C–H asymmetric (2924 cm^−1^) and symmetric (2852 cm^−1^) stretches further confirms hydrosilylation, linking the ligand to the ncSi surface. Following quaternization, we observe the appearance of CH_3_ symmetric stretching (2979 cm^−1^) and N–C asymmetric stretching (970 cm^−1^) modes attributed to quaternized ligands.

X‐ray photoelectron spectroscopy (XPS) was conducted to further evaluate the state of the alkyl halide ligand before and after quaternization. In Figure 2b,c, the Br 3d peak is shown to shift to lower binding energies after the quaternization reaction, which indicates a gain in electron density upon conversion from an alkyl bromide to a bromide anion. The high‐resolution XPS scan for ncSi:C11Br in the Br 3d region shows a single peak at 70.62 eV, consistent with literature C–Br binding energy values.^36^ Postquaternization (Figure 2c), we observed a shift in binding energy to 69.87 and 68.81 eV, which is characteristic of bromide anions. The broadness of the bromide peak is due to a range of binding energies, which correspond to various positions where the bromide anions reside relative to the cationic head groups. A similar change in binding energy should also be true for the N 1s orbital but due to its low sensitivity, no prominent peak was observed in the relevant binding energy region.^37^, ^38^ Survey XPS scans can be found in Figure S4 (Supporting Information) showing signals characteristic of ncSi and the FTO substrate.

### 2.3 Assessing the Surface Charge of Quaternary Ammonium‐Passivated ncSi

**Figure 3:**
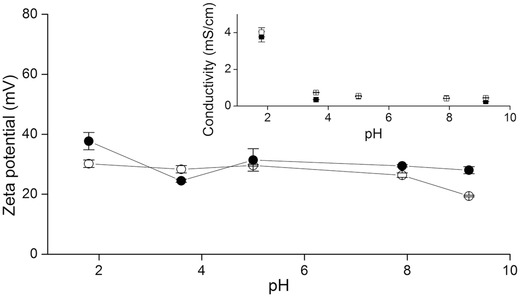
Zeta potential and conductivity (inset) of ncSi:C11TMA (closed shapes) and ncSi:C6TMA (open shapes) samples at various pH levels.

A distinctive and important property of quaternary ammonium‐functionalized ncSi is the retention of positive surface charge regardless of solution pH. This can be seen by the average measured zeta potential values of ncSi:C11TMA and ncSi:C6TMA, which were relatively stable at 30.2 ± 4.4 mV and 26.8 ± 3.9 mV, respectively, between pH 1.8 and 9.2 (**Figure** **3**). Note that the higher conductivity observed for the pH 2 sample was due to the increased concentration of HCl, which surpasses that of the minimum ionic strength used for the other samples (10 × 10^−3^ m NaCl). On average, there is no significant difference in zeta potential between the six‐carbon ligand of ncSi:C6TMA and eleven‐carbon ligand of ncSi:C11TMA, hence it would be difficult to conclude whether one ligand allows for more surface charge over another. When ncSi:C11Br is reacted with triethylamine instead of trimethylamine, the resulting triethylammonium cation‐functionalized SiNC showed poor dispersibility in water with low zeta potential (≈5 mV). This is due to the added steric hindrance of the charged atom by the ethyl groups, preventing hydrophilic interaction with water.

### 2.4 Size‐Dependent Photoluminescence of Quaternary Ammonium‐Functionalized ncSi

**Figure 4:**
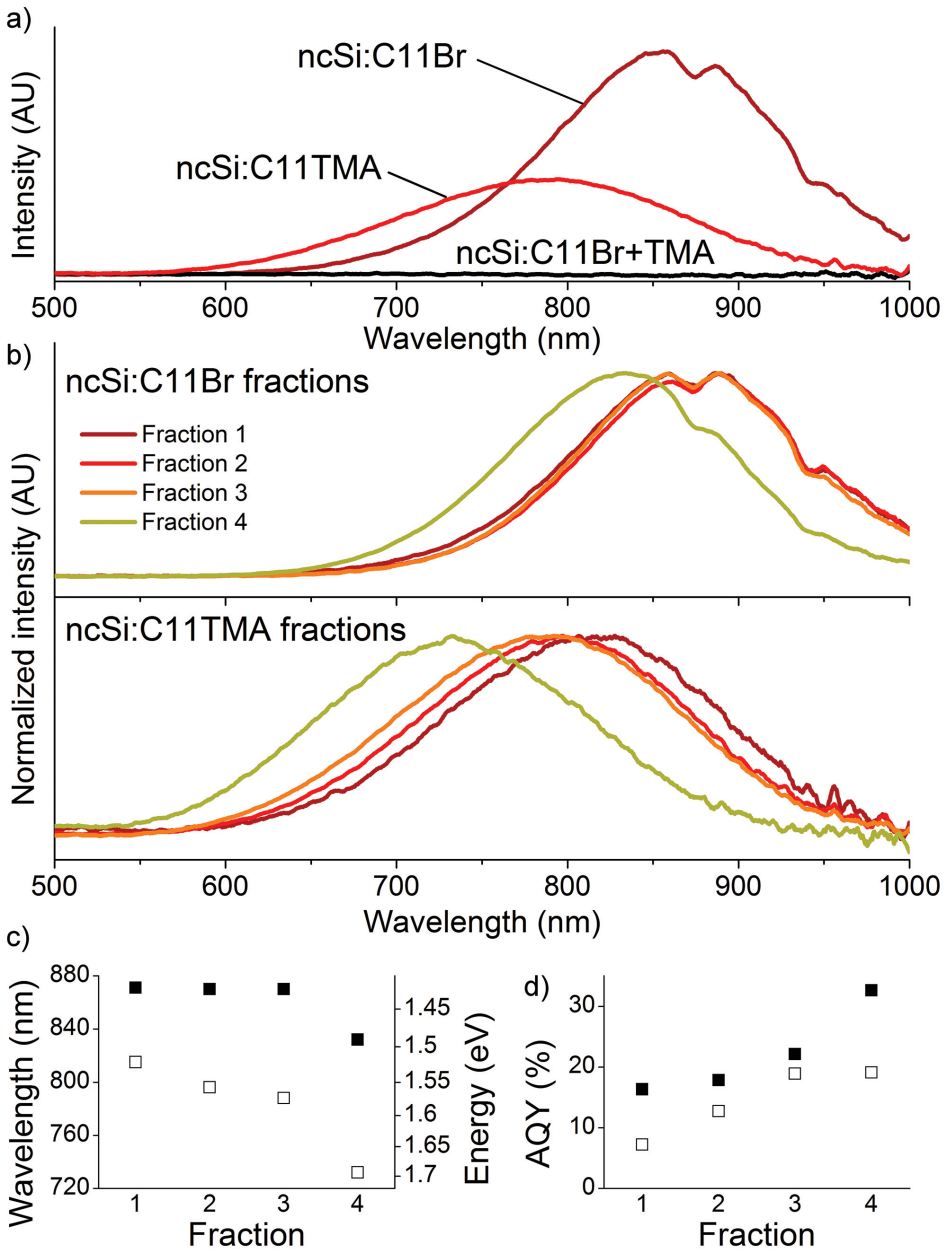
The PL spectra a) of ncSi:C11Br, ncSi:C11Br with TMA, and ncSi:C11TMA after treatment with TFA, showing the quenching effect of TMA and anti‐quenching effect of TFA. b) PL spectra, c) PL maxima and d) PL AQY of size‐separated fractions of ncSi:C11Br before (closed squares) and after (open squares) quaternization and purification show the effects of etching and oxidation during the reaction.

The ability to emit light in the 650–900 nm spectral region is critical for biological fluorescent labeling applications. Excess TMA in the quaternization reaction form dative‐bonded adducts with silicon, which effectively quench the PL of the ncSi.^27^, ^29^ In order to reduce the PL‐quenching effect of the TMA adducts, TFA was used to protonate the TMA groups and form trimethylammonium trifluoroacetate, which can then be removed by washing in water. This quenching and anti‐quenching effect is shown in **Figure** **4**a, where ncSi:C11Br shows a PL maximum centered at 859 nm and no PL is observed after the addition of TMA. After quaternization with TMA, subsequent treatment with TFA reverses the quenching effect and reveals a PL peak centered at 789 nm. The blue‐shift in the PL maximum can be attributed to a combination of etching effects by hydroxides from trace water in the strongly basic TMA solution and oxidation effects over time.^25^ Etching reduces the size of the ncSi, whereas oxidation consumes surface silicon species to form a silicon oxide layer. Both phenomena effectively increase the bandgap resulting in a blue‐shift in PL emission due to quantum‐confinement effects near the Bohr exciton radius of silicon (≈5 nm).^13^ Another effect of the formation of a silicon oxide layer is the creation of surface trap states and defect sites, which decrease the ensemble PL absolute quantum yield (AQY) from 18.0% to 11.7%. TFA treatment of TMA adducts have been shown to only partially restore the PL, hence it is also possible that remnant TMA adducts contribute to the lowered AQY of the product. Overall, the AQY are consistent with literature values of similar alkyl‐passivated ncSi populations in the same size range with high size‐dispersity, which suffer from averaging effects due to sizes with lower AQY.^35^

While SiNC:C11TMA displays stable PL emission during post‐synthesis purification steps, SiNC:C6TMA does not (Figure S5, Supporting Information). Simple washing procedures using ethanol and deionized water caused rapid quenching in the PL of ncSi:C6TMA attributed to the formation of surface Si–OH moieties, which lead to irreversible surface oxidation preventing radiative recombination.^25^ The presence of these Si–OH groups is shown in the FTIR spectra of both six‐carbon samples, ncSi:C6Br and ncSi:C6TMA (Figure S6, Supporting Information), indicating that the shorter alkyl chain does not protect the ncSi surface against oxidation as well as the longer alkyl chain of ncSi:C11Br and ncSi:C11TMA.^39^ Indeed, only a small Si–OH peak is observed in the FTIR spectra of ncSi:C11TMA despite similar purification conditions.

PL emission of quantum‐confined ncSi is attributed to core excited‐state transitions and which scale with particle dimensions. PL mediated by surface excited‐state transitions are not as strongly affected by particle dimensions as much as they are by surface chemistry.^30^ To show that the synthesized quaternary ammonium functionalized ncSi emit PL via a core‐mediated pathway subject to quantum‐confinement effects, we have size‐separated the high‐dispersity ncSi:C11Br sample into low‐dispersity populations by size‐selective precipitation.^33^ This technique has been successfully applied by many researchers to successfully size‐separate fractions of nanoparticles by exploiting their ligand interaction with the solvent system.^25^, ^35^, ^40^, ^41^, ^42^ As the solvent polarity is gradually altered by addition of a poor solvent for the ncSi, particles of larger size and surface ligand amount will precipitate sooner before smaller particles. Each size‐separated sample was then independently reacted with TMA and treated with TFA followed by PL measurement (Figure 4b).^43^

As expected, the fractions show a decreasing trend in PL maxima with decreasing population size (or increasing fraction number, Figure 4c). This effect is less pronounced in the ncSi:C11Br fractions due to the low number of fractions made in order to conserve yield for subsequent characterization. Within each fraction, particles with higher AQY contribute more to the spectral peak, resulting in peaks skewed towards these high QY populations. In our case, the smaller, lower wavelength particles have higher AQY, hence an averaging effect is seen for the first three fractions towards ≈870 nm. After quaternization, the ncSi:C11TMA fractions show a more pronounced difference likely due to increased surface oxidation of smaller particles relative to larger particles due to their larger surface‐to‐volume ratio. These results are consistent with analogous alkyl‐ or 10‐undecenoic‐acid‐terminated ncSi systems previously published.^12^

The AQY displayed an increasing trend with decreasing size consistent with previous studies on ncSi at this size and emission range (Figure 4d). In a recent study by Sun et al., the relationship between ncSi size and PL properties was discussed in depth.^35^ AQY was found to initially increase as particle size decreases before reaching a maximum of ≈19% at 800 nm (≈3.8 nm diameter) beyond which it decreases as the increased surface‐to‐volume ratio and surface organic‐ligand and surface‐defect‐mediated non‐radiative vibrational relaxation begin to overwhelm the rate of radiative recombination due to quantum confinement. The ensemble ncSi:C11Br and ncSi:C11TMA have ≈4.8 nm diameter, with PL maxima between 800 and 900 nm hence they fall into the first regime where AQY increases with decreasing particle size.

Postquaternization, the PL maxima changed from 871–832 nm to 815–732 nm. The difference in PL maxima increased with decreasing fraction size from 0.10 to 0.20 eV, this is due to an increasing surface‐to‐volume ratio, which renders it more susceptible to oxidation and etching induced blue‐shift in PL. The AQY changed from between 16.3% and 32.6% to 7.2% and 19.1%. If the ncSi:C11TMA fractions were emitting PL based on a surface‐mediated mechanism, it is expected that both the PL maxima and AQY will center at one value independent of sample size. However, since we observe clear decreasing trends in PL maximum and increasing trends in AQY, we believe that the product is emitting via a core‐mediated mechanism despite the presence of amines in the system.

## 3 Conclusion

In this report, we have described the synthesis of terminal alkyl halide‐functionalized ncSi, which can be size selectively separated into low‐dispersity populations, and which emit PL in the NIR‐to‐red spectral range. We have further demonstrated their flexibility as a chemical precursor by forming colloidally stable quaternary ammonium‐functionalized ncSi, which exhibit unprecedented pH‐independent positive surface charge, while preserving its optical properties even in the presence of amines. This novel class of quaternary ammonium‐ functionalized ncSi may find applications in biological fluorescent labeling where solution stability, pH‐independent surface charge, small particle hydrodynamic diameter, and NIR‐to‐red PL are desired properties. Looking to the future, opportunities now abound for studying electrostatic self‐assembly phenomena of size and surface controlled silicon nanocrystal cations and anions analogous to those recently pioneered with gold and silver nanoionic systems.^44^

## 4 Experimental Section

*Materials and Methods*: Silicon monoxide (SiO, −235 mesh powder) was used as received. Dichloromethane (ACS grade; Sigma–Aldrich), ethanol (anhydrous, Commercial Alcohols), hexanes (ACS grade, Caldon), hydrofluoric acid (ACS 48 wt% aqueous solution, Sigma–Aldrich), methanol (ACS grade, Fisher Scientific), trifluoroacetic acid (99%, Sigma–Aldrich), trimethylamine in ethanol (31–35 wt%; Sigma–Aldrich), toluene (Anhydrous, Caledon), and 6‐bromo‐1‐hexene (98%, Oakwood Chemical) were used as received. Triethylamine (99.5%, Sigma–Aldrich) and 11‐bromo‐1‐undecene (96%; Alfa Aesar) was distilled under vacuum prior to use. Dimethyl sulfoxide (DMSO, ACS grade, ACP) was dried under 4Å molecular sieves prior to use.

*Preparation of Hydride‐Capped Silicon Nanocrystals (ncSi:H)*: 1.5 g of SiO powder was transferred into the center of a quartz reaction boat, taking care to minimize spread, and placed in a LINDBERG high temperature tube furnace flushed with a stream of 5% hydrogen in argon gas. Due to temperature gradients which form in the tube furnace, minimizing spread of the reagent helps maintain uniform heat treatment of the sample. The dark brown powder was then heated at a rate of 18 °C min^−1^ until a peak temperature of 925 °C where the temperature was held constant for 0.5 h before cooling naturally under constant gas flow. Once cooled, a dark brown powder of ncSi in SiO_2_ matrix was collected and stored under ambient conditions. A sample of the dark brown powder (0.3 g) was transferred to a polypropylene beaker equipped with a magnetic stir‐bar. 10 mL of ethanol was used to wet the dark brown composite. Hydrofluoric acid (48 wt%, 20 mL) was then added to the mixture and stirred for 2 to 6 h to etch away the SiO_2_ matrix and reduce the size of the nanocrystal. The ncSi:H was extracted from the solution by liquid–liquid extraction using hexanes (5 mL) into a 35 mL microwave reaction tube equipped with a magnetic stir‐bar and septum. The ncSi:H solution was distilled at room temperature using a Schlenk vacuum line to remove DCM.C_6_

*Synthesis of ncSi:C11Br and ncSi:C6Br*: The dried ncSi:H was purged with N_2_ gas and 3 mL of 11‐bromo‐1‐undecene (3.19 g, 13.67 mmol) or 2 mL 6‐bromo‐1‐hexene (2.44 g, 14.96 mmol) was added to produce a cloudy brown solution. The solution was then degassed and purged with N_2_ gas three times over the course of 30 min. The ncSi:H in neat 11‐bromo‐1‐undecene was placed in a CEM Discover SP temperature controllable microwave reactor with variable output from 0–200 W at 2450 MHz. The sample was irradiated in the 35 mL reaction tube for 1 h with a maximum temperature setting of 180 °C. The average power during reaction was approximately 50 W. A translucent dark brown solution of surface‐functionalized ncSi:C11Br or ncSi:C6Br in excess ligand was obtained. The solution was transferred to a 15 mL centrifuge tube. 10 mL methanol and 0.5 mL ethanol were added to precipitate the nanoparticles and the solution was centrifuged at 7800 rpm (6461 g) for 10 min. The supernatant of 11‐bromo‐1‐undecene, methanol, and ethanol was decanted and stored to be distilled to retrieve the unreacted ligand. The pellet was re‐dispersed in ethanol (13 mL), sonicated, and centrifuged again in a washing procedure repeated three times to remove trace amounts of unreacted free ligand. The final pellet was re‐dispersed in toluene or ethanol for storage.

*Synthesis of ncSi:C11TMA and ncSi:C6TMA*: ncSi:C11Br or ncSi:C6Br in DMSO (4 mL) was added to a 35 mL microwave reaction tube equipped with a magnetic stir‐bar and trimethylamine in ethanol (1 mL, 4.2 mmol). The solution was irradiated in a microwave reactor for 1 h with a maximum temperature setting of 80 °C to form a translucent light brown solution. Trifluoroacetic acid (2.98 g, 26.1 mmol) was added to form trimethylammonium trifluoroacetate salt. The solution was stirred for 48 h, then washed with 2:1 ethanol/hexanes (10 mL) and deionized (DI) water (3 × 10 mL) by repeated centrifugation and re‐dispersion by sonication.

*Size‐Selective Precipitation of ncSi:C11Br*: 3 mL of ncSi:C11Br in 11‐bromo‐1‐undecene solution (as synthesized) was transferred to a 15 mL centrifuge tube and to it, 0.5 mL ethanol and 1 mL methanol was added. The cloudy solution was centrifuged at 7800 rpm (6461 g) for 10 min. The supernatant was transferred to another centrifuge tube and the process repeated to produce a series of fractions with decreasing size. The pellets were washed with 2:1 ethanol/hexanes (3 × 10 mL) by repeated centrifugation and re‐dispersion by sonication.

## Supporting information

As a service to our authors and readers, this journal provides supporting information supplied by the authors. Such materials are peer reviewed and may be re‐organized for online delivery, but are not copy‐edited or typeset. Technical support issues arising from supporting information (other than missing files) should be addressed to the authors.

Supplementary
