# Supplementary material for: Cationic Silicon Nanocrystals with Colloidal Stability, pH‐Independent Positive Surface Charge and Size Tunable Photoluminescence in the Near‐Infrared to Red Spectral Range
Source: Adv Sci (Weinh). 2016 Jan 21;3(2):1500263. doi: 10.1002/advs.201500263 (PMC5066801; doi:10.1002/advs.201500263)
Supplement: Supplementary file 1 — Supplementary [file ADVS-3-0j-s001.pdf]

Copyright WILEY-VCH Verlag GmbH & Co. KGaA, 69469 Weinheim, Germany, 2016.

ADVANCED SCIENCE
Open Access

Supporting Information

for *Adv. Sci.*, DOI: 10.1002/advs. 201500263

Cationic Silicon Nanocrystals with Colloidal Stability, pH-Independent Positive Surface Charge and Size Tunable Photoluminescence in the Near-Infrared to Red Spectral Range

*Kenneth K. Chen, Kristine Liao, Gilberto Casillas, Yiying Li, and Geoffrey A. Ozin**

Copyright WILEY-VCH Verlag GmbH & Co. KGaA, 69469 Weinheim, Germany, 2013.

Supporting Information

Cationic Silicon Nanocrystals with Colloidal Stability, pH Independent Positive Surface Charge and Size Tunable Photoluminescence in the Near-Infrared to Red Spectral Range

*Kenneth K. Chen, Kristine Liao, Gilberto Casillas, Yiying Li, and Geoffrey A. Ozin**

Characterization of ncSi:C11TMA and ncSi:C6TMA

Zeta potential was measured in 10 mM NaCl with pH adjusted using 0.1 M NaOH or HCl. Samples of ncSi:C11TMA or ncSi:C6TMA in ethanol (2 mg/mL) were dispersed in pH-adjusted solutions for a final concentration of 0.1 mg/mL and measured using a Malvern Zetasizer Nano system in a disposable zeta dip cell. The data was processed using Malvern Zetasizer software adjusting for solvent conditions and using the Smoluchowski approximation.

Attenuated Total Reflectance Fourier Transform Infrared (ATR-FTIR) spectra were measured between 550 and 4000 cm-1 on a Perkin Elmer Spectrum One spectrometer with ATR attachment and processed using Perkin Elmer Spectrum software. Samples of ncSi:C11TMA or ncSi:C6TMA were precipitated by centrifugation and dried under vacuum before measurement.

Photoluminescence (PL) spectra and absolute photoluminescence quantum yield (APLQY) were measured in the range of 300 – 1000 nm with excitation at 365 nm. Samples of ncSi:C11TMA or ncSi:C6TMA were dispersed in 800 µL ethanol. Measurements were conducted using an Oceanoptics Maya2000 spectrometer and processed using in-house LabVIEW software. Detailed description on the calculation of APLQY can be found in a previous publication.[1]

X-ray photoelectron spectroscopy (XPS) data were obtained using a Thermo Scientific Theta Probe utilizing monochromatic Al K α radiation, and spectra were calibrated to the C1s emission arising from adventitious hydrocarbons (284.8 eV). Samples were drop cast from ethanol or toluene solution onto FTO substrate.

Scanning transmission electron microscopy (STEM) images were acquired in a probe-corrected JEOL ARM200F operated at 80 kV equipped with a cold field emission gun and a high resolution pole-piece. Middle angle annular dark field (MAADF) images were acquired with 45 and 180 mrad inner and outer collection angles respectively, while BF images used 11 mrad collection angles. Both images were recorded with a dwell time of 20 μs, and convergence semi-angle of 25 mrad resulting in a probe current of 40 pA. Samples were drop-casted onto Graphene-Cu grids (Graphene Supermarket) to ensure an ultra-thin support film of less than 2 nm.

Supplementary Figures

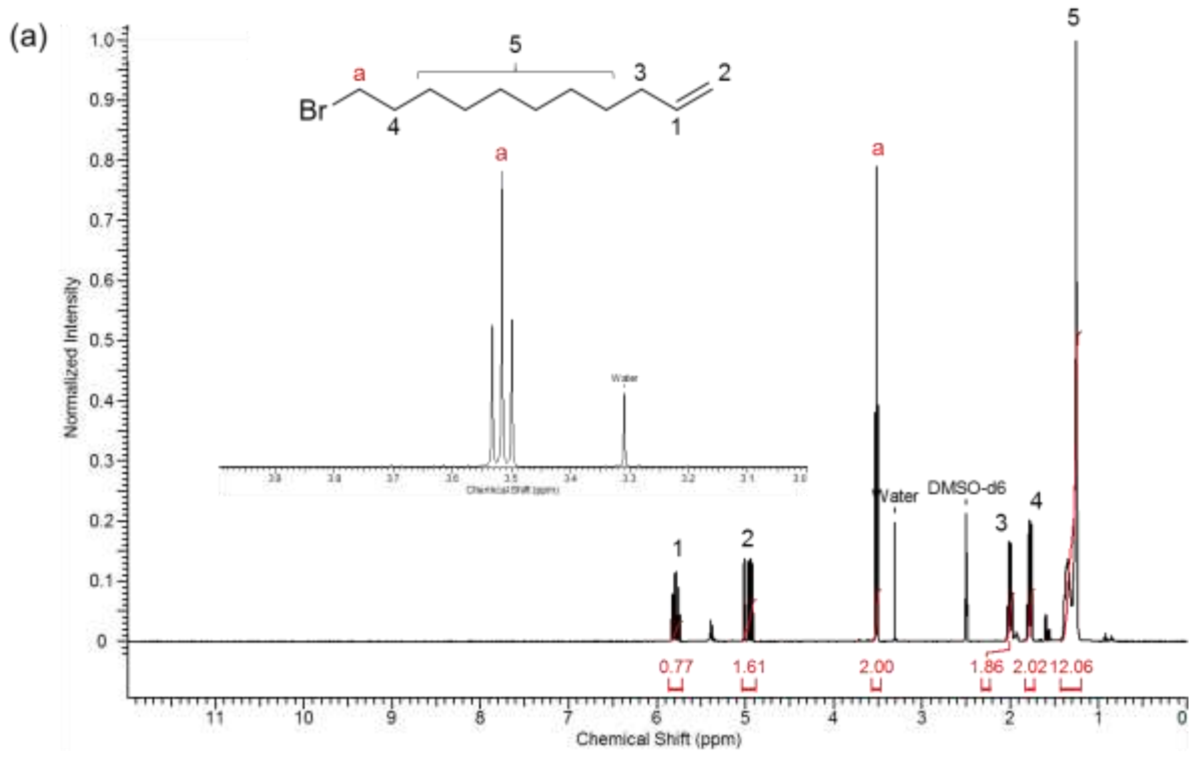

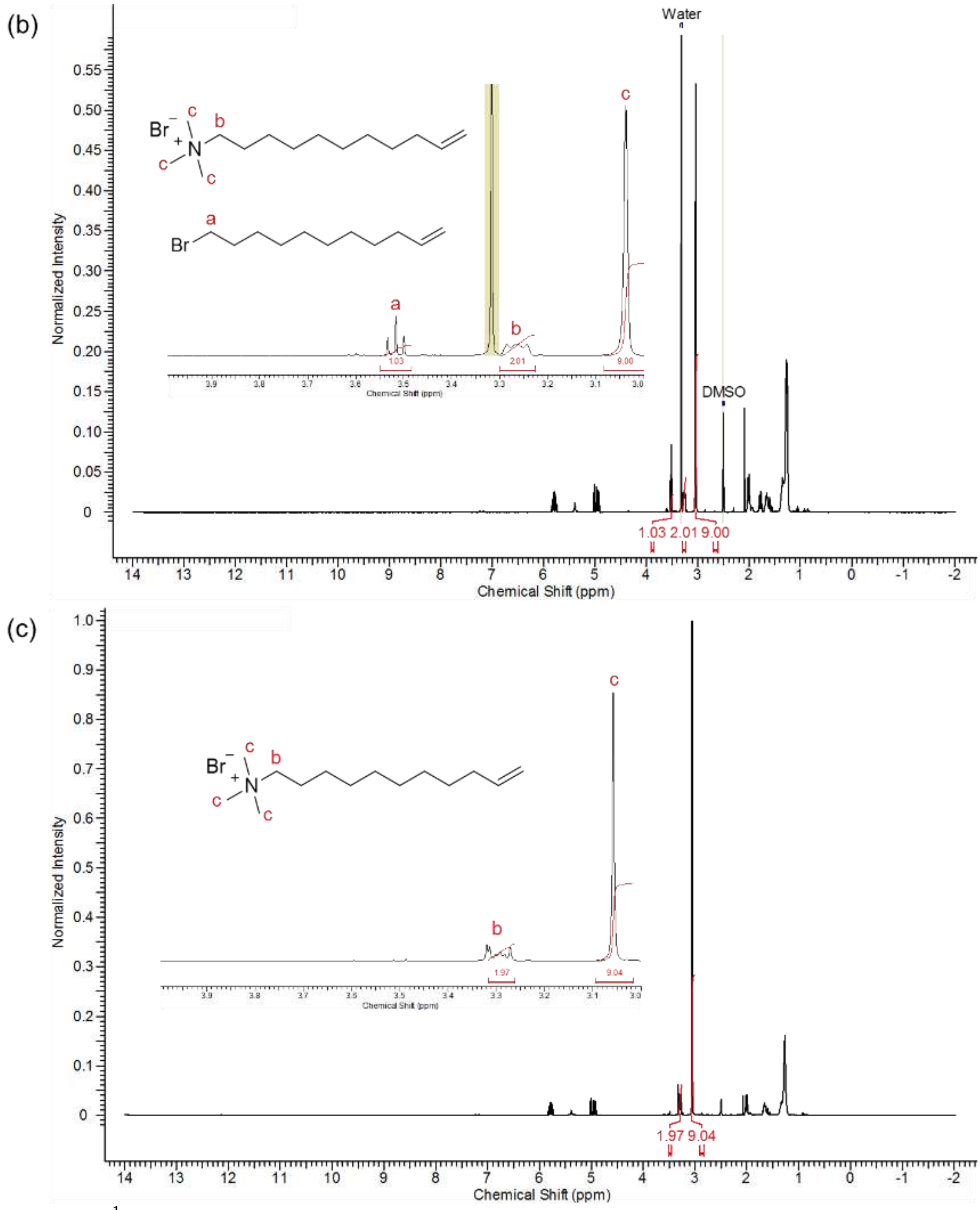

Figure S1. ^{1}H NMR spectra of C11Br after 0 min. (a), 5 min. (b), and 45 min. (c) microwave reaction at 80 °C with TMA. Insets show 3 – 4 ppm region with significant peaks labeled. The inset in (c) confirms complete quaternization by 45 min., therefore the 1 h reaction time used for ncSi:C11Br should lead to near-complete quaternization.

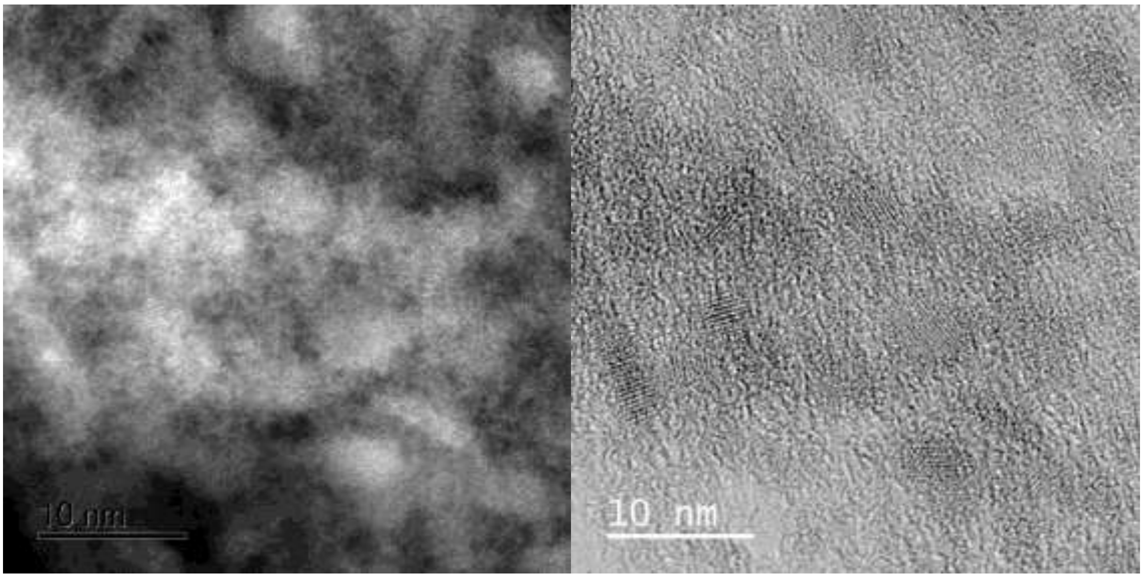

Figure S2. MAADF-STEM (left) and BF STEM (right) images of ncSi:C11TMA. Particles with lattice fringes can be seen in both images, their 0.31 nm lattice constants are indicative of ncSi.

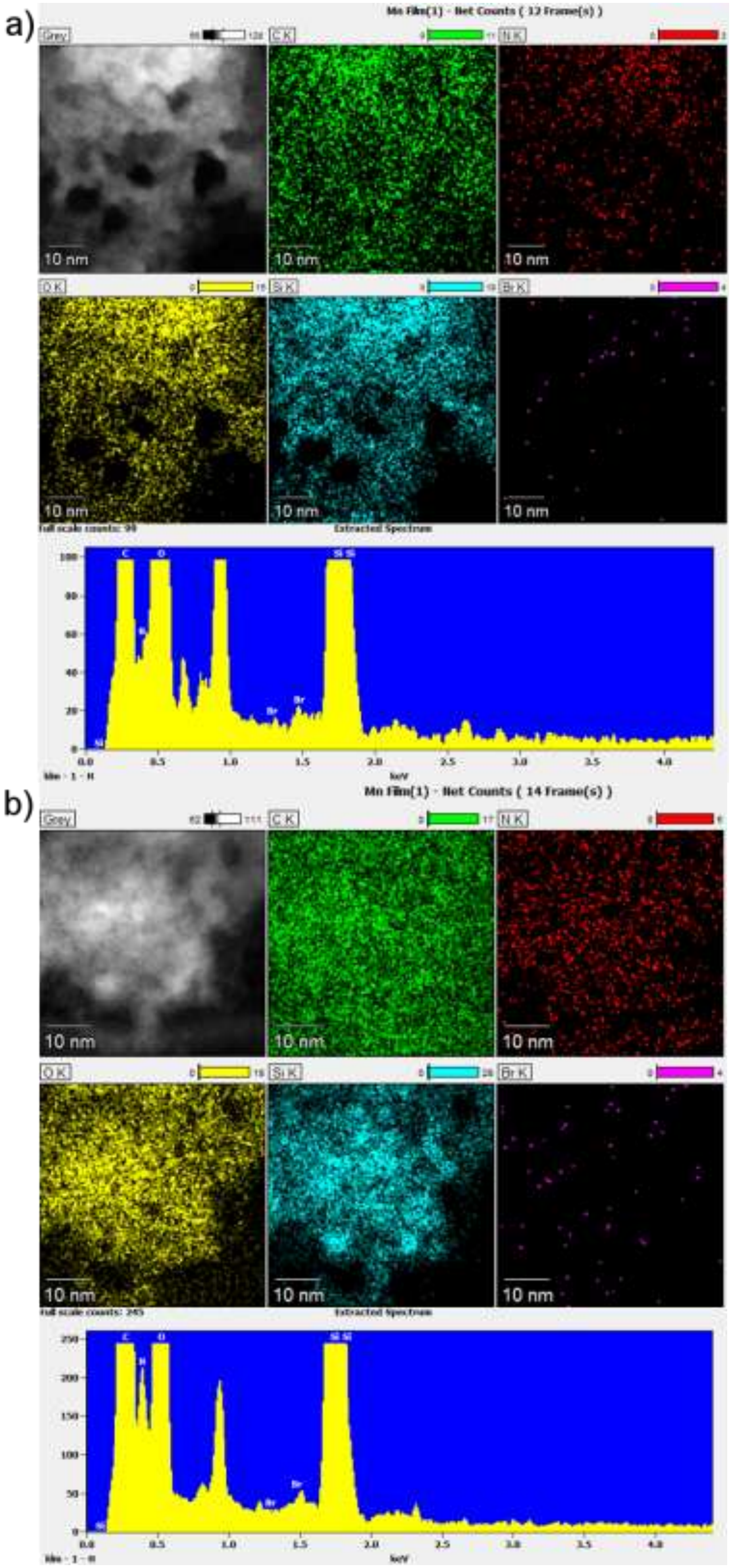

Figure S3. EDX images and spectra of ncSi:C11Br (a) and ncSi:C11TMA (b) samples. The insets shows the MAADF-STEM image and signals corresponding to carbon, nitrogen, oxygen, silicon and bromine sources from left to right, top to bottom. The spectra at the bottom shows clear Si, C, O and Br signals in both situations, the N signal peak is more significant after quaternization.

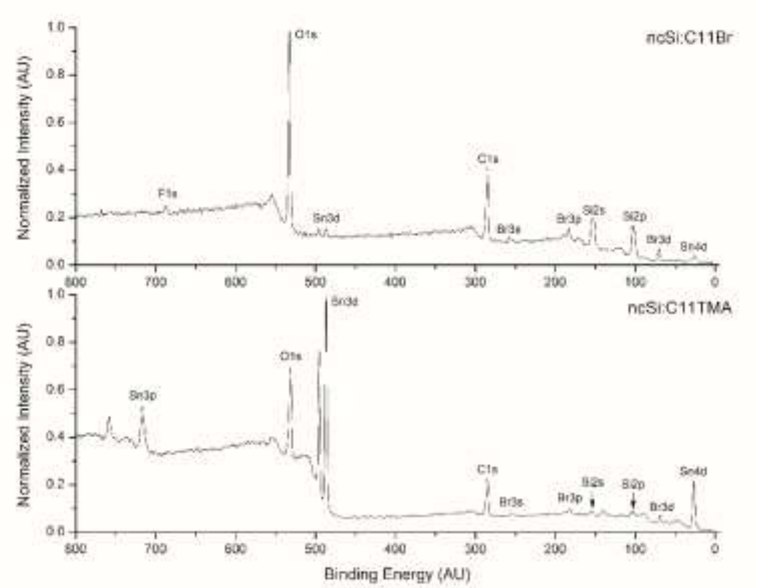

Figure S4. Survey XP spectra of ncSi:C11Br (top) and ncSi:C11TMA (bottom). Signals corresponding to Si, O, C and Br are shown, while signal for N are below the sensitivity limit.

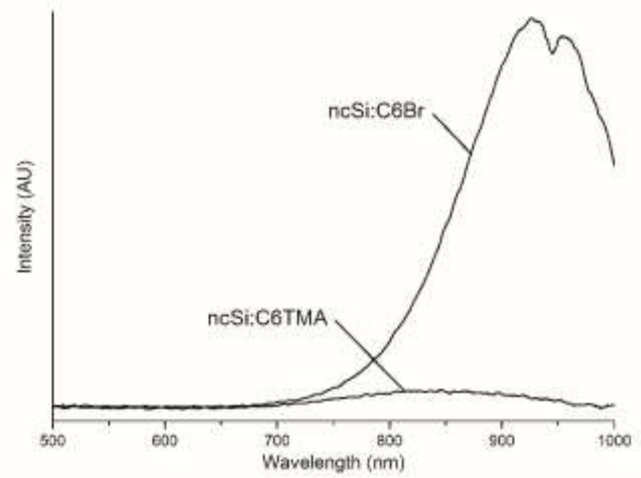

Figure S5. PL spectra of ncSi:C6Br and ncSi:C6TMA. The measured AQY decreases from 17.6% to 0.9% due to the formation of surface oxides.

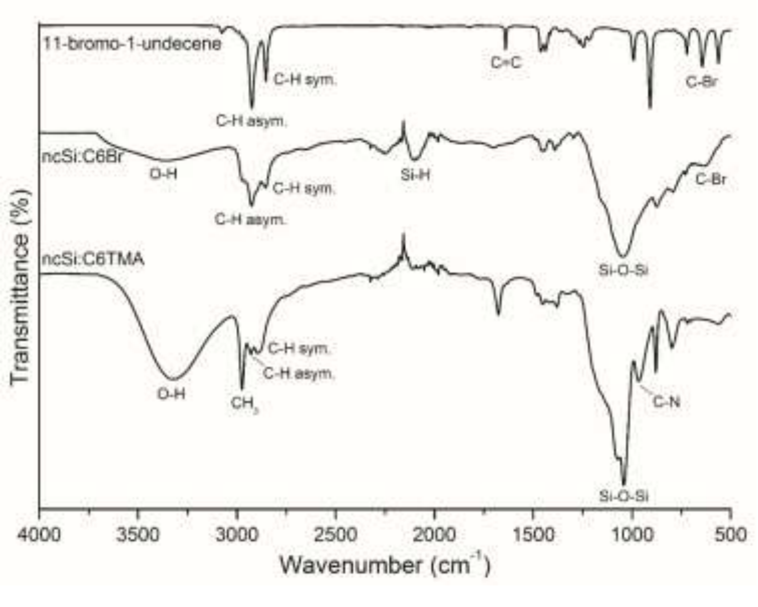

Figure S6. ATR-FTIR spectra of 11-bromo-1-undecene, ncSi:C6Br, and ncSi:C6TMA (from top to bottom) with significant modes labeled. The significant oxidation which occurs during quaternization is evidenced by the prominenet OH peak formed.

References

1 D. O. Faulkner, J. J. McDowell, A. J. Price, D. D. Perovic, N. P. Kherani, G. A. Ozin, *Laser Photon. Rev.* **2012**, *6*, 802.
